# Unveiling Macrosomia Risks of Non-Diabetic Women: Insights from Second Trimester Maternal Lipid Profiles

**DOI:** 10.34172/aim.31914

**Published:** 2024-11-01

**Authors:** Ahkam Göksel Kanmaz, Yasemin Alan, Murat Alan, Emrah Töz

**Affiliations:** ^1^Department of Obstetrics and Gynecology, Health Science University Tepecik Training and Research Hospital, Izmir, Türkiye; ^2^Department of Obstetrics and Gynecology, Eşrefpaşa Hospital, Izmir, Türkiye

**Keywords:** Fetal growth, Macrosomia, Maternal lipid profile, Non-diabetic pregnant women, Prenatal care, Triglycerides

## Abstract

**Background:**

Macrosomia, characterized by excessive fetal growth, is common in infants born to women with pre-gestational diabetes and gestational diabetes mellitus (GDM). However, macrosomia, which leads to birth-related maternal and fetal complications and metabolic complications in the adolescence of the affected fetuses, also occurs in the pregnancies of non-diabetic women. This study aims to identify the association between second-trimester lipid profiles and macrosomia in non-diabetic pregnant women to aid in early diagnosis.

**Methods:**

This retrospective cohort study included 8,956 patients who delivered at a tertiary care center between 2017 and 2019. Exclusion criteria encompassed pre-existing diabetes, GDM, preeclampsia (PE), intrahepatic cholestasis of pregnancy, obesity, fetal chromosomal or genetic abnormalities, tobacco, alcohol, or drug use affecting lipid metabolism. Participants were divided into two groups: 621 with macrosomia and 873 controls. Second trimester maternal lipid profiles and demographic variables such as age, pregnancy week, and gender were assessed.

**Results:**

In the study cohort, maternal age (*P*=0.002), gestational week (*P*=0.003), and cesarean section rate (*P*<0.001) were higher in the macrosomic group. High-density lipoprotein-cholesterol (HDL-C) was significantly lower, while total cholesterol (TC), triglycerides (TG), and low-density lipoprotein-cholesterol (LDL-C) were significantly higher in the macrosomic group (*P*<0.001). Univariate analysis revealed positive associations between second-trimester TG (OR 1.023, 95% CI: 1.020‒1.033, *P*<0.001), TC (OR 1.023, 95% CI: 1.016‒1.030, *P*<0.001) and LDL-C (OR 1.036, 95% CI: 1.018-1.054, *P*<0.001) with macrosomia and a negative association with HDL-C (OR 0.954, 95% CI: 0.923‒0.976, *P*<0.001). However, after adjusted multivariable logistic analysis, only TG remained statistically significantly associated with macrosomia (OR 1.054, 95% CI: 1.033‒1.076, *P*<0.001).

**Conclusion:**

Our study emphasizes the importance of early recognition and prevention of macrosomia. Structured prospective studies are needed to enhance macrosomia prediction and implement preventive measures, such as dietary modifications. These strategies will be crucial in preventing birth-related complications and long-term health risks, including diabetes, obesity, and cardiovascular diseases, associated with macrosomia.

## Introduction

 Birth weight is influenced by a range of maternal factors, including gestational age, parity, maternal age, body mass index (BMI), racial background, smoking status, and medical history.^1,2^ Macrosomia, characterized by excessive fetal growth, is typically diagnosed when the birth weight exceeds the 90th percentile limit for gestational age at 40 weeks of pregnancy (or 4000 g).^3^ This condition is anticipated in infants born to pregnant women with pre-gestational diabetes and gestational diabetes mellitus (GDM), as well as non-diabetic pregnant women.^4-6^ The prevalence of fetal macrosomia has remained stable over time, as reported in a study by Hamisu M. Salihu in the United States.^7^ Globally, epidemiological data indicate prevalence rates ranging from 0.5% to 14.9%, with an 8.6% prevalence rate of macrosomia among non-diabetic pregnant women reported in Turkey.^7-10^

 Elevated birth weight can be associated with maternal complications such as prolonged labor and increased operative births, including cesarean sections and perineal tears.^11^ Infants with macrosomia are at risk of birth-related injuries such as brachial plexus injury and shoulder dystocia, as well as metabolic imbalances like early-onset hypoglycemia and hypomagnesemia. Moreover, macrosomia predisposes children to long-term health risks, including diabetes, obesity, and cardiovascular diseases.^4,12-15^

 Macrosomia can result from a combination of genetic and environmental factors, leading to metabolic alterations *in utero*. Fetal overgrowth is primarily driven by the fetus’s utilization of amino acids and lipids, which are metabolized through maternal glucose intake.^16-18^ During pregnancy, changes in lipid metabolism in maternal-fetal physiology are influenced by hyperlipidemia. In non-diabetic women, the lipid-related risk for macrosomia may be more closely tied to maternal adiposity and the effects of lipids on placental nutrient transfer. Optimal maternal lipid profiles contribute to favorable conditions for fetal development, with elevated triglycerides (TGs) increasing fetal glucose supply and low-density lipoprotein cholesterol (LDL-C) influencing placental steroidogenesis. These metabolic alterations in the intrauterine environment contribute significantly to fetal weight gain.^19,20^ A significant association has been identified between second-trimester maternal serum lipid profiles and the risk of macrosomia, with evidence indicating a positive correlation.^21^ Although hyperglycemia plays a central role in driving fetal overgrowth in non-diabetic mothers, the relationship between lipid profiles and macrosomia remains underexplored. Furthermore, it continues to be debated which specific lipid parameters are most strongly associated with macrosomia.

 Identifiable factors in mid-pregnancy, particularly for non-diabetic mothers, may serve as valuable indicators for preventing potential complications associated with macrosomia. Interventions such as dietary and lifestyle modifications for non-diabetic mothers at risk of macrosomia could help prevent the condition from manifesting.

 Therefore, the aim of our study is to investigate changes in lipid profiles as a potential risk factor and to analyze the correlation between maternal lipid levels and macrosomia in uncomplicated pregnancies.

## Materials and Methods

###  Study Population 

 This retrospective cohort study was conducted among 8,956 patients who delivered at our tertiary care center between 2017 and 2019. The study started after the approval of the Tepecik Training and Research Hospital Ethics Committee (2019/17-2). The study cohort comprised patients without pre-existing type 1 or type 2 diabetes mellitus, GDM, preeclampsia (PE), or intrahepatic cholestasis of pregnancy, and those who were not obese either before or during pregnancy. Patients with fetal chromosomal or genetic abnormalities diagnosed either prenatally or postnatally were excluded. All patients for whom the file could not be accessed through the hospital information system and who had missing data that could potentially affect the analysis results were excluded from the study. Additionally, those who used tobacco, consumed alcohol, or took drugs affecting blood lipid metabolism during pregnancy were also excluded.

 Initial antenatal assessments for the patients were conducted at our clinic. During their first trimester prenatal visits, the participants’ BMI and waist circumference were measured, and their income and educational background categories were assessed. Subsequently, lipid profile analyses were performed during the second trimester. The results from these assessments and tests were sourced from the hospital information system. Throughout the pregnancy follow-ups, any missing data were cross-checked and added to the database via the Ministry of Health’s prenatal care system. For those who delivered at our clinic, delivery records and neonatal assessments (Apgar scores, genetic abnormalities, etc.) were verified and documented through the hospital information system.

 During the analysis conducted between 2017 and 2019, a total of 1494 participants (16.6%) who met the inclusion criteria were included out of 8956 patients. All participants included in the study were categorized into two groups based on birth weight: the macrosomic group, which included 621 participants, and the control group, consisting of 873 non-macrosomic participants.

###  Biochemical Analysis 

 Venous blood samples were collected from all participants during the second trimester of pregnancy (24–26 gestational weeks) following an overnight fast to conduct the oral glucose tolerance test (OGTT) and assess lipid profiles. Each sample underwent comprehensive biochemical analyses to measure total cholesterol (TC), TG, high-density lipoprotein cholesterol (HDL-C), LDL-C, and glucose levels. All biochemical analyses adhered to standardized laboratory protocols. Lipid measurements were conducted using automated biochemical analyzers (Olympus AU5400, Tokyo, Japan and Beckman Coulter AU 5800). The calibration of automated biochemical analyzers in our hospital is conducted at least once a year, in accordance with the Regulation on Testing, Control, and Calibration of Medical Devices published in the Official Gazette on May 25, 2015, number 29397 by the Ministry of Health.

###  Statistical Analysis

 Statistical analysis was performed using the SPSS 26.0 software (SPSS Inc., Chicago, Illinois). The Shapiro-Wilk and Kolmogorov-Smirnov tests were utilized as normality tests, suitable for the sample size for all continuous variables. Additionally, normal distribution was verified using Q-Q plots and histograms. Normality tests were conducted, and parametric variables were presented as mean ± standard deviation and analyzed using the independent *t* test, and the assumption of homogeneity of variance was assessed using Levene’s test. Non-parametric variables were assessed using the Mann-Whitney U-test, with results expressed as median (minimum, maximum). Categorical variables were analyzed using the chi-square test or Fisher’s exact test, depending on the number of variables. Logistic regression analysis was utilized to investigate the relationships between maternal dyslipidemia and macrosomia. The multivariable adjusted model included maternal age, gestational age at birth and fetal sex which were found statistically significant with macrosomia in the study population regarded as confounding variables. A *P* value < 0.05 was considered statistically significant.

## Results

###  Demographic Characteristics 

 Following the application of inclusion and exclusion criteria to our cohort, 621 (6.9%) patients met the criteria for the macrosomic group and 873 patients for the control group. Maternal and neonatal demographic data are presented in Table 1. In the macrosomic group, age (*P* = 0.002), nulliparity (*P* = 0.011), gestational week (*P* = 0.003), and cesarean section rate (*P*< 0.001) were found to be statistically higher. Although HDL-C was significantly lower in the macrosomic group (*P* < 0.001), other lipid levels such as TC, TG, and LDL-C were statistically higher (*P* < 0.001). Additionally, the proportion of male infants was higher in the macrosomic group (*P* = 0.012), despite the statistically lower Apgar score at 5 minutes (*P* = 0.026).

**Table 1:** Demographic Data and Pregnancy Outcomes of the Pregnant Women Included in the Study

	**Non-macrosomic (n=873)**	**Macrosomic (n=621)**	***P *** **Value**
**Maternal Findings**			
Age	30.3 ± 5.6	31.1 ± 4.3	0ç000ç002 0.0026
BMI	27.1 (23.5‒29.4)	28.6 (20.4‒29.8)	0.159
Waist circumference, cm	77 (60-98)	79 (73-90)	0.799
Parity			0.011
Nulliparous	213 (24.4%)	188 (30.2%)	
Multiparous	660 (75.6%)	433 (69.8%)	
Maternal educational background			0.319
Primary-secondary education	703 (80.5%)	487 (79.1%)	
Higher education	170 (19.5%)	134 (20.9%)	
Maternal income level			0.366
Low income	407 (46.6%)	324 (52.1%)	
High income	473 (53.4%)	297 (47.9%)	
Gestational week	39 (34‒40)	40 (38‒40)	0.003
Birth weight	3510 (2400‒4000)	4360 (4050‒4680)	< 0.001
Mode of delivery			< 0.001
Vaginal	737 (84.4%)	468 (75.3%)	
Cesarean section	136 (15.6%)	153 (24.7%)	
TG	105.8 ± 54.04	204.42 90.5	< 0.001
TC	182.52 ± 33.2	224 ± 50.4	< 0.001
HDL	65.91 ± 14.1	58.8 ± 17.96	< 0.001
LDL	105.5 ± 27.3	125.2 51.8	< 0.001
**Fetal Findings**			
Male infant	373 (42.7%)	306 (49.2%)	0.012
APGAR 1-min	7 (3‒7)	7 (5‒7)	0.824
APGAR 5-min	8 (5‒9)	7 (7‒8)	0.026

TG, Triglyceride; TC, Total cholesterol; HDL, High-density lipoprotein; LDL, Low-density lipoprotein

###  Associations Between Maternal Lipid Profile and Macrosomia

Table 2 demonstrates a positive association between second-trimester TG (OR 1.023, 95% CI: 1.020-1.033,* P* < 0.001), TC (OR 1.023, 95% CI: 1.016‒1.030,* P* < 0.001) and LDL-C (OR 1.036, 95% CI: 1.018‒1.054, *P* < 0.001) with macrosomia and a negative association with HDL-C (OR 0.954, 95% CI: 0.923‒0.976, *P* < 0.001). However, after multivariable analysis, it was found that the statistically significant relationship between LDL-C and TC and macrosomia was no longer present. The only remaining statistically significant positive association was between TG and macrosomia (*P* < 0.001, OR 1.026, 95% CI: 1.016‒1.031) and the negative association between HDL-C and macrosomia persisted (*P* < 0.001, OR 0.976, 95% CI: 0.965‒0.981). A multivariable logistic regression analysis was conducted, adjusting for maternal age, gestational age at birth, and fetal sex (Table 3). The only lipid parameter that remained significant was TG. Elevated TG levels were associated with an increased risk of macrosomia, with an adjusted odds ratio (aOR) of 1.054 (95% CI: 1.033‒1.076, *P* = 0.001).

**Table 2:** Associations Between Maternal Lipid Profile and Macrosomia

	**Univariate Analysis**		**Multivariate Analysis**	
	**Unadjusted OR (95% CI)**	***P *** **Value**	**Unadjusted OR (95% CI)**	***P *** **Value**
TG	1.023 (1.020‒1.033)	< 0.001	1.026 (1.016‒1.031)	< 0.001
TC	1.023 (1.016‒1.030)	< 0.001	1.005 (990‒1.019)	0.518
HDL	0.954 (0.923‒0.976)	< 0.001	0.976 (0.965‒0.981)	0.001
LDL	1.036 (1.018‒1.054)	0.001	0.995 (0.979‒1.011)	0.537

TG, Triglyceride; TC, Total cholesterol; HDL, High-density lipoprotein; LDL, Low-density lipoprotein; OR, Odds ratio.

**Table 3:** Macrosomia Associated with Lipid Profile in Multivariate Logistic Model*

	**Unadjusted OR**	**95% Confidence Interval OR**	***P *** **Value**
TG	1.054	1.033‒1.076	< 0.001
TC	1.014	0.983‒1.046	0.375
HDL	0.992	0.971‒1.034	0.160
LDL	1.023	0.986‒1.060	0.224

*Adjusted for maternal age, gestational age at birth and fetal sex. TG, Triglyceride; TC, Total cholesterol; HDL, High-density lipoprotein; LDL, Low-density lipoprotein; OR, Odds ratio.

## Discussion

 This study has identified a significant correlation between elevated maternal lipid levels, particularly TG, during the second trimester of pregnancy, and macrosomia in non-diabetic mothers. The results serve as a starting point for the development of plans aimed at preventing macrosomia, which can lead to numerous maternal, birth-related, and long-term health risks for children.

 In early pregnancy, lipid levels decline within the first 6 weeks but steadily rise until the third trimester. This shift signifies an initial accumulation of fat depots in first trimester, transitioning to an active breakdown of adipose tissue later in pregnancy.^21,22^ These changes in lipid metabolism during pregnancy play a significant role in the development of fetal fat mass in addition to glucose, which serves as a crucial nutrient crossing the placenta alongside amino acids.^21^

 Our study reveals a strong positive correlation between maternal triglyceride concentrations and the risk of macrosomia among non-diabetic pregnant women. Triglycerides, which are associated with birth weight and postnatal growth, have been reported to be initially transported to the yolk sac and then to the fetus as pregnancy progresses, in conjunction with placental function from the early weeks of pregnancy.^23^ Xi et al^24^ identified maternal TG as an independent predictor of macrosomia in non-diabetic patients, albeit in a smaller cohort. Additionally, several recent publications have reported a positive correlation between elevated TG levels and the risk of macrosomia.^18,25^ Therefore, our results are consistent with the idea that triglycerides are effective in predicting macrosomia.

 HDL-C, essential for embryonic development due to its role in intrafollicular cholesterol homeostasis,^16^ exerts protective effects through its anti-oxidative and anti-inflammatory properties in physiological states.^22^ In our study, a negative correlation was observed between HDL-C levels and the risk of macrosomia. Similarly, several studies have reported associations between low HDL-C concentrations and pregnancy complications.^21,23^ As noted by Wang et al,^24^ evaluating HDL-C concentration is crucial for assessing fetal growth.

 Our study initially suggested a link between TC and LDL levels with macrosomia, but these associations did not hold up in analyses. Similarly, Zhu et al^25^ reported a positive relationship between first-trimester TC levels and birth weight, but they found no significant connection between TC and LGA or macrosomia in their overall or subgroup analyses. In the same vein, Shi et al observed no association between second-trimester TC or LDL levels and adverse outcomes like macrosomia.^22^ These findings are consistent with previous research and align with the results of our study.^23,26,27^

 The retrospective nature of this study and the fact that blood lipid parameters were only measured in the second trimester are limitations. However, the study’s strengths include the inclusion of only non-GDM patients and adherence to inclusion criteria to minimize the impact of potential demographic differences on the results.

 The main limitation of this study is its retrospective design, which restricts access to missing data. Consequently, the exclusion of patients with incomplete data has resulted in a reduction of our cohort size. Furthermore, since the patients included in the study were non-diabetic, lack of regular blood glucose monitoring and reliance on BMI values recorded in the hospital information system may represent biases that we were unable to mitigate. Despite all limitations, the inclusion criteria of our study largely mitigated potential biases and minimized the impact of demographic differences on the results. Additionally, our findings support the role of lipid parameters as potential markers for predicting macrosomia in non-diabetic mothers.

 Fetal macrosomia is typically identified only after development through prenatal ultrasound. The primary goal should be early recognition and prevention of macrosomia. This study is significant as it highlights the possibility of identifying macrosomia before it occurs, even in pregnancies without risk factors. Our study is important as it provides insights for future research targeting macrosomia prediction and serves as both a source of data and a potential starting point. Structured prospective studies aimed at enhancing macrosomia prediction and implementing preventive measures, such as dietary modifications, will be crucial in averting birth related complications and long-term health risks such as diabetes, obesity, and cardiovascular diseases stemming from macrosmia.

## Conclusion

 This study emphasizes the significance of maternal lipid levels, particularly triglycerides and HDL-C, in predicting macrosomia in non-diabetic pregnancies. The findings highlight a potential pathway for early identification of fetal overgrowth risk, even in pregnancies that lack other established risk factors. By recognizing these lipid markers in the second trimester, clinicians can adopt preventive approaches, potentially including dietary and lifestyle modifications, to mitigate adverse birth outcomes associated with macrosomia. Future research should focus on prospective studies that explore maternal lipid levels throughout pregnancy, aiming to refine prediction models and develop targeted interventions to minimize complications related to fetal macrosomia, thereby reducing associated long-term health risks like diabetes, obesity, and cardiovascular diseases in offspring.
